# Endotoxin Contamination in Nanomaterials Leads to the Misinterpretation of Immunosafety Results

**DOI:** 10.3389/fimmu.2017.00472

**Published:** 2017-05-08

**Authors:** Yang Li, Mayumi Fujita, Diana Boraschi

**Affiliations:** ^1^Department of Dermatology, University of Colorado, Anschutz Medical Campus, Aurora, CO, USA; ^2^Institute of Protein Biochemistry, National Research Council (CNR), Napoli, Italy

**Keywords:** engineered nanomaterials, immunosafety assessment, endotoxin contamination, endotoxin evaluation, Limulus amebocyte lysate assay

## Abstract

Given the presence of engineered nanomaterials in consumers’ products and their application in nanomedicine, nanosafety assessment is becoming increasingly important. In particular, immunosafety aspects are being actively investigated. In nanomaterial immunosafety testing strategies, it is important to consider that nanomaterials and nanoparticles are very easy to become contaminated with endotoxin, which is a widespread contaminant coming from the Gram-negative bacterial cell membrane. Because of the potent inflammatory activity of endotoxin, contaminated nanomaterials can show inflammatory/toxic effects due to endotoxin, which may mask or misidentify the real biological effects (or lack thereof) of nanomaterials. Therefore, before running immunosafety assays, either *in vitro* or *in vivo*, the presence of endotoxin in nanomaterials must be evaluated. This calls for using appropriate assays with proper controls, because many nanomaterials interfere at various levels with the commercially available endotoxin detection methods. This also underlines the need to develop robust and bespoke strategies for endotoxin evaluation in nanomaterials.

## Introduction

Nanotechnology has undergone a rapid growth all over the world, with the production of a broad array of different nanomaterials in many consumers’ products, to which the human population and the environment are therefore increasingly exposed. The health and environmental impacts of these new engineered nanomaterials (ENM) are a topic of considerable interest for nanotech industries and regulators as well as scientists, leading to the attempt of building safe-by-design ENM and the effort of establishing clear and relevant safety guidelines (1). Among nanotoxicity effects, induction of inflammation is considered a risk-predictive key effect (2). Several ENM were found to trigger inflammation in experimental models both *in vitro* and *in vivo*, suggesting a possible risk for human health (3–7). However, many experimental studies that show inflammatory effects triggered by ENM did not properly consider the possible presence of endotoxin. The Gram-negative endotoxin or lipopolysaccharide (LPS) is a ubiquitous contaminant in our environment and a potent inducer of inflammation and cell death. Hence, when evaluating the toxic and inflammatory effects of ENM to establish their safety, we must be aware that the presence of endotoxin in ENM can lead to inaccurate findings and consequently misleading conclusions (8).

Endotoxin/LPS is a molecule found in the outer membrane of Gram-negative bacteria and consists of a hydrophilic polysaccharide domain and a hydrophobic lipid domain. LPS plays an important role in bacterial virulence, because of its lipid part (lipid A) responsible for cytotoxicity. In mammalian tissues, LPS binds to a soluble LPS-binding protein, which transports LPS to the cell surface receptor, Toll-like receptor (TLR) 4. TLR4, together with MD2 and CD14, initiates signaling that leads to activation of inflammation pathways in different cell types (9). Because TLR4 is expressed by many cells, in particular innate immune cells such as monocytes and macrophages, these cells are very sensitive and responsive to LPS stimulation and raise a defensive inflammatory response against bacterial infections (10). LPS-activated cells produce and secrete a great number of inflammatory factors including interleukin (IL)-1β, IL-6, IL-8, and tumor necrosis factor-α. At high concentrations, LPS can also directly kill cells, although it depends on cell sensitivity. Given its potent inflammatory/toxic activity, exposure to endotoxin can induce serious and even life-threatening effects, including respiratory symptoms, asthma, and endotoxemia (11–14). Therefore, the acceptable endotoxin levels in medical products (such as surgical instruments or drugs) have been regulated by US FDA as early as 1985, updated thereafter, and accepted/adopted almost all over the world (15). Pharmaceutical companies must follow these regulations, and the presence of endotoxin in medical use products or intravenous (i.v.) drugs must be certified to be below a given limit before their release in the market. However, this regulation does not apply to ENM that are not intended for medical use, meaning that most industrially produced ENM are not screened for endotoxin contamination. While this may not be a health problem unless the ENM are administered i.v. into human beings, it still remains a relevant issue because the results from extremely sensitive nanosafety models used for assessing products’ safety may be biased by the presence of contaminating endotoxin and reveal inflammatory/toxic effects that are not ENM specific but rather endotoxin dependent.

## Endotoxin Contamination of Nanomaterials

Endotoxin is a thermoresistant molecule that can persist in the environment in the absence of live Gram-negative bacteria. Its thermostability makes endotoxin resistant to the routine sterilization methods applied in biology laboratories (16). Thus, endotoxin is a ubiquitous environmental contaminant, present in all chemicals and glassware used in laboratories (17). Special attention or treatment is needed for avoiding/eliminating endotoxin contamination, which includes working in endotoxin-free conditions and depyrogenation of materials. A common and effective method for depyrogenation is incineration, which implies dry heating of tools and materials at high temperatures for given times, e.g., 180°C for 3 h or 250°C for 30 min (18). However, these extreme conditions are not suitable for depyrogenating most ENM, because the treatment may change the ENM physicochemical properties. França et al. used different methods (UV irradiation, gas–plasma treatment, ethylene oxide treatment, formaldehyde treatment, and autoclaving) for sterilizing/depyrogenizing two differently sized gold (Au) nanoparticles (NPs). They found that the various methods caused changes in the Au NPs, the most common problem being NPs aggregation and consequent changes in UV–Vis spectra, morphology, and particle size distribution. They further tested the biological effects of these Au NPs and found that the different sterilization procedures could affect the NPs cytotoxic capacity and their ability to induce intracellular ROS (19). Hence, the best way to obtain endotoxin-free ENM is to take precautions and synthesize them in endotoxin-free conditions (20). As most chemical labs and manufactures do not apply particular precautions, the ENM undergoing nanosafety and preclinical nanomedicine efficacy studies are likely to get contaminated by endotoxin. Furthermore, ENM have a large reactive surface area, which tends to absorb molecules from the surrounding milieu to reduce its energy, thereby facilitating the adsorption of surface contaminants (21). The lipid domain allows endotoxin attachment to hydrophobic surfaces, while the negatively charged phosphate groups promote endotoxin interaction with cationic surfaces (22). In addition, coordinative binding can occur between the negatively charged LPS and loosely anionic surfaces (e.g., citrate-coated Au NPs), resulting in firm and stable binding (23).^1^ Therefore, endotoxin can attach to virtually any surface, which makes endotoxin a common contaminant for many different kinds of ENM (8, 21). Darkow and coworkers have shown that functionalized NPs could bind endotoxin through Coulomb and van der Waals interactions (24). Bromberg et al. showed a strong interaction between lipid A (the toxic moiety of endotoxin) and functionalized paramagnetic ENM (25). The capacity of endotoxin to bind with NPs was also observed for polystyrene particles (26). Our recent study showed that endotoxin binds to the surface of Au NPs in a dose-dependent manner (23). Abadeer et al. studied the role of surface properties in the interaction of Au nanorods with endotoxin by using surface plasmon resonance sensing and found that endotoxin attaches more easily to a cationic surface compared to neutral or anionic surfaces (27). Our data with Au NPs indeed confirm that the ENM surface characteristics can affect the binding of endotoxin (23, see text footnote 1).

**Figure 1:**
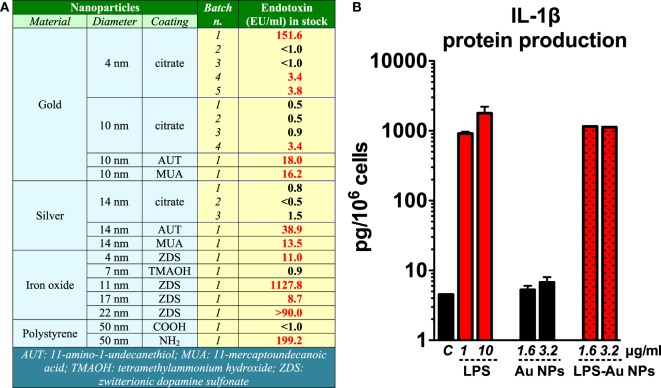
**Endotoxin contamination in nanoparticles (NPs) induces inflammatory effects**. **(A)** Endotoxin contamination in different nanomaterials evaluated by Limulus amebocyte lysate assay. **(B)** Gold (Au) NPs were deliberately contaminated with 1 µg/ml lipopolysaccharide (LPS) for 1 h at room temperature and then thoroughly washed with endotoxin-free water to eliminate unbound LPS. Human primary monocytes were exposed to either endotoxin-free or endotoxin-coated Au NPs for 24 h. The production of interleukin (IL)-1β in the culture supernatants was measured by ELISA [data partially presented in the supporting material of Ref. (30)].

We have lab tested several commercial ENM or ENM received from collaborators and found variable degrees of endotoxin contamination (unpublished data; Figure 1A). In a study in which NPs synthesis was repeated in normal conditions or after glassware and tool depyrogenation, we could show that taking precautions could significantly dampen the endotoxin contamination in ENM (28). On the other hand, a heavy endotoxin contamination in polystyrene ENM after long-term storage (over 6 months) may have been due to the poor handing processes (29). Thus, we should be aware that endotoxin contamination in ENM is a common phenomenon.

## Biological Effects of Endotoxin-Contaminated Nanomaterials

The biological effects of endotoxin-contaminated ENM have been reviewed recently (8). Endotoxin-carrying ENM can initiate the TLR4 signaling pathway in innate immune cells, activate the inflammasome, and induce the secretion of IL-1β, a fundamental cytokine that plays an important role in physiological and pathological conditions (31), as well as many other inflammation-related factors. We have shown that the endotoxin bound on the surface of Au NPs turned those NPs from inactive to highly inflammatory and able to induce secretion of IL-1β in human primary monocytes (Figure 1B) (30). With this in mind, many reports that show inflammatory and toxic effects of ENM *in vitro* or *in vivo* on TLR4-expressing cells need to be taken with caution if the endotoxin level was not assessed. Studies have shown that ENM can activate a TLR4-dependent inflammatory response in the target cells. Some of these studies failed to assess or did not mention the potential contamination of the ENM under study with endotoxin (32, 33), which makes it impossible to assess the reliability of the results. On the other hand, other studies showed the ability of ENM to initiate TLR4-dependent activation in the absence of measurable endotoxin contamination or by excluding the effects of endotoxin [see, for instance, Ref. (34)], thereby suggesting a *bona fide* ENM effect. Qu et al. reported that graphene oxide can be sensed by TLR4 and induce macrophage necrosis through the caspase-3 pathway (35). Endotoxin was measured in this study with a Limulus amebocyte lysate (LAL) endpoint chromogenic kit from Lonza and declared to be about 0.1 EU/ml (1 ml containing 80 µg of NPs). This brings us to another concern, i.e., the possible interference of graphene oxide with the LAL assay. Indeed, interference has been extensively reported for many ENM (36–39), which strongly suggests the need for testing the interference for each ENM under investigation. In addition, the Lonza QCL-1000 endpoint chromogenic LAL assay with readout at 405 nm has been shown to be unsatisfactory for measuring endotoxin in metal and metal oxide (39) as well as graphene oxide ENM (40). With all this in mind, we conclude that not only should we measure endotoxin in ENM but also we must make sure that the endotoxin detection assay is reliable and relevant to the ENM under study. Without the formal proof of the absence of endotoxin contamination, the *bona fide* bioeffects of ENM cannot be accurately assessed.

## Endotoxin Evaluation Methods in Nanomaterials

The FDA-approved methods to detect endotoxin are the rabbit pyrogen test (RPT) as an *in vivo* test and the LAL assay as an *in vitro* test. Alternative and sensitive bioassays are also approved by the European Centre for the Validation of Alternative Methods (ECVAM) for assessing pyrogens, such as the human PBMC activation assay and the human monocytes activation test (MAT). However, the RPT *in vivo* assay and *in vitro* bioassays using PBMC and monocytes are not specific for endotoxin, because they measure inflammatory effects (induction of fever and induction of inflammatory cytokines) and thus detect responses from all types of inflammation-inducing agents (which may include EMN). Therefore, to specifically detect the endotoxin level in ENM, the LAL assay is recommended.

The LAL assay could provide fast, sensitive, and specific endotoxin assessment. The only other molecule that gives a positive result with the traditional LAL assay is β-glucan, which, however, can be inhibited by a specific buffer in the currently available commercial LAL kits. Because of its specificity, sensitivity, and reliability, the LAL assay has replaced the old *in vivo* RPT as the assay chosen by all regulatory agencies, such as FDA, European, Chinese, and Japanese pharmacopeias (41–44). The use of the LAL assay for endotoxin detection in ENM is also regulated by ISO29701:2010 regulation “Nanotechnologies—Endotoxin test on nanomaterial samples for *in vitro* systems” (45).

In the LAL assay, factor C, an enzyme derived from the amebocytes of the horseshoe crab *Limulus polyphemus*, is activated by exposure to endotoxin and in turn induces activation of a clotting enzyme. Based on the types of detection of the clotting enzyme activity, three variants of the LAL assay are commercially available, including the gel clot, the turbidimetric, and the chromogenic assays. Recently, using recombinant factor C instead of the *Limulus* amebocyte lysate, new fluorescence-based assays have been developed. These assays have the advantage of being totally specific for endotoxin, because β-glucan activates factor G but not factor C. Although the LAL assay can reliably detect endotoxin in soluble reagents, the physicochemical characteristics of ENM pose a significant problem of interference with both the components and the detection readouts (fluorescence, optical density) of various assays (28, 36, 39). To overcome the interference problem, the available assays need to be validated for the lack of interference by ENM with the catalytic activity of the enzyme(s), substrate cleavage, and the final readout signals (8, 39). It has been shown that the gel clot LAL assay is not accurate for testing endotoxin contamination in particles, while the chromogenic LAL assay showed higher sensitivity and no interference (46). The unsuitability of the gel clot assay has also been shown for silica, silver (Ag), titanium dioxide, calcium carbonate, and other clinical-grade NPs (37, 38, 47), suggesting that the gel clot assay should not be used for testing endotoxin in ENM in general. However, despite these new evidences, the use of the gel clot assay is still recommended in a FDA guidance document to solve discrepancies between results from different LAL formats in industry (48). Furthermore, our results with the chromogenic LAL assay suggested that metal and metal oxide NPs may interfere with the final readout by absorbing the final dye (p-nitroaniline) and quenching the readout, leading to underestimating the endotoxin contamination (39). Therefore, Dobrovolskaia et al. have declared that none of the currently available LAL formats is optimal for endotoxin assessment in ENM and suggested that at least two LAL formats with different endpoints/readouts should be used. The results should also be confirmed by RPT when the LAL results show more than 25% difference (36, 38). This approach has been used at the Nanotechnology Characterization Laboratory of the National Cancer Institute (USA) for measuring the endotoxin contamination in ENM.

The bioassays, on the other hand, may be adequate to assess pyrogenic/inflammatory effects in general, in particular for the ENM for clinical use. These bioassays (RPT *in vivo* and PBMC and MAT *in vitro*) are not specific for endotoxin, since they are based on the development of an inflammatory response (e.g., fever, NF-ĸB activation, secretion of inflammatory cytokines), which can be induced by any kind of pyrogen, theoretically including ENM. Therefore, bioassays cannot distinguish between effects induced by endotoxin and other pyrogens and intrinsic effects of ENM. The use of the PBMC or the MAT tests in parallel to the LAL assay should allow us to detect, in addition to endotoxin, the possible presence of other pyrogenic agents, which may be present but cannot be detected with the LAL assays. Thus, Dobrovolskaia et al. suggested to use such assays to confirm the LAL results (38). We have tested endotoxin contamination in Au, Ag, and iron oxide (Fe_3_O_4_) NPs with the chromogenic LAL assay of Associates of Cape Cod (endpoint readout at 540 nm) and in parallel with the ECVAM-approved PBMC activation assay (IL-6 production) (39). The endotoxin contamination detected by the LAL assay was confirmed by the PBMC activation assay only for Au NPs, but not for Ag and Fe_3_O_4_ NPs. This is probably due to the interference of NPs with some elements in the bioassay. Most likely, the NPs interfere with the ELISA-based IL-6 detection process by interfering with antigen/antibody interaction, adsorbing and subtracting IL-6, or quenching the optical signal that indicates the presence of IL-6. Thus, the biological assays also need an accurate characterization and validation before their results can be used to detect endotoxin in ENM. Table 1 summarizes the pros and cons of different endotoxin evaluation methods for EMN.

**Table 1:** **Advantages and disadvantages of assays used to detect endotoxin**.

	Limulus amebocyte lysate (LAL) assay					Bioassay	
	Traditional			Modified		Rabbit pyrogen test	*In vitro* activation assay
	Gel clot	Turbidimetric	Chromogenic	Fluorogenic	EndoLISA		
Pros	Short-term experiment and easy performance, specific for endotoxin, most used endotoxin measurement methods					Most relevant assays for pyrogen detection can be used to screen nanomedicine for preclinic usage	

	Easy and cheap	Quantitative, high sensitivity	Quantitative, high sensitivity, two different detection wavelengths	High sensitivity, very specific (no recognition of β-glucan)	Washing steps can eliminate interfering substances compared to other LAL assays, wide endotoxin detection range
	
Cons	Semiquantitative, low sensitivity, prone to subjective variations, not precise, proved to be interfered by nanoparticles (NPs)	Due to their turbidity, high optical density NPs or NPs at high concentration may interfere with this assay	Can be interfered by NPs with absorbance at or close the detection wavelength (405 or 540 nm)	NPs may interfere with enzyme reaction or quench fluorescence	NPs may interfere with lipopolysaccharide (LPS) antibody binding. Not clear if washing could detach LPS (bound to the wells) from particles, or remove LPS from wells together with particles, or leave LPS-coated particles in the wells. Residual particles in wells, if not washed off, may interfere with enzyme reaction or quench fluorescence	Non-specific for endotoxin, reactive to any inflammation-inducing agent (including some NPs). The interference of NPs with these assays still needs accurate evaluation	
						Animal usage, high cost, low sensitivity	NPs may induce cytotoxicity and interfere with cell activation *in vitro*. NPs may also interfere with the ELISA procedures used for detecting inflammatory factors (e.g., antibody–antigen binding, color development, optical readouts)
	
Use	NPs interference should be predetermined (e.g., turbidity, the optical interference). Appropriate procedures could be also applied to overcome interference, such as dilution or switching to another detection wavelength. Additional controls should be run to exclude interference with assay components (e.g., measuring endotoxin recovery rate)					Can be applied in combination with the LAL assay for analyzing the parenteral drugs (nanodrug) during the earlier development phase. Generally used when different LAL assays show >25% variation. However, interference of NPs with bioassays may prevent from solving the problem					
	
	Final decision is usually made based on the gel clot assay in industry. This regulation is, however, unsuitable for NPs because of their significant interference with the assay	Applied to NPs that interfere with the chromogenic assay	Commonly used assay in biology labs. Can be used with NPs after appropriate controls	May be used for NPs that do not have autofluorescence and do not quench fluorescence	Use for NPs should be accurately validated (see Cons above)

## Conclusion and Future Perspective

To reliably assess safety of ENM, either intended for medical use or included in commercial products, it is important to take into careful consideration the presence of unwanted bioactive contaminants, of which bacterial endotoxin is most common and abundant. This would eliminate misinterpretation of experimental results and erroneous attribution to ENM of toxic effects that may be entirely due to contaminants. Thus, nanosafety/nanomedicine researchers and regulators should be aware of the possible contamination of ENM with highly inflammatory contaminants such as endotoxin and design and adopt appropriately designed assays. Likewise, chemists/producers should design their synthesis processes to minimize endotoxin contamination. Furthermore, since the methods for endotoxin assessment in ENM are still challenging (Table 1) and the regulations on nanoproducts are still incomplete, robust strategies and bespoke assays need to be developed for endotoxin evaluation in ENM.

## Author Contributions

YL wrote the paper, MF revised it, and DB contributed to writing and critically revised it.

## Conflict of Interest Statement

The authors declare that the research was conducted in the absence of any commercial or financial relationships that could be construed as a potential conflict of interest.

## Footnotes

^1^Li Y, Shi Z, Radauer-Preiml I, Andosch A, Casals E, Luetz-Meindl U, et al. Bacterial Endotoxin (LPS) Binds to the Surface of Gold Nanoparticles, Interferes with Biocorona Formation and Induces Human Monocyte Inflammatory Activation.
